# Association of healthy lifestyle with incident cardiovascular diseases among hypertensive and normotensive Chinese adults

**DOI:** 10.3389/fcvm.2023.1046943

**Published:** 2023-03-02

**Authors:** Jian Su, Houyue Geng, Lulu Chen, Xikang Fan, Jinyi Zhou, Ming Wu, Yan Lu, Yujie Hua, Jianrong Jin, Yu Guo, Jun Lv, Pei Pei, Zhengming Chen, Ran Tao

**Affiliations:** ^1^Department of Noncommunicable Chronic Disease and Prevention, Jiangsu Provincial Center for Disease Control and Prevention, Nanjing, China; ^2^Department of Epidemiology, School of Public Health, Nanjing Medical University, Nanjing, China; ^3^Department of Noncommunicable Chronic Disease Control and Prevention, Suzhou City Center for Disease Control and Prevention, Suzhou, China; ^4^Wuzhong District of Suzhou City Center for Disease Control and Prevention, Suzhou, China; ^5^Fuwai Hospital Chinese Academy of Medical Sciences, Beijing, China; ^6^Department of Epidemiology and Biostatistics, School of Public Health, Peking University, Beijing, China; ^7^Peking University Center for Public Health and Epidemic Preparedness and Response, Beijing, China; ^8^Clinical Trial Service Unit & Epidemiological Studies Unit, Nuffield Department of Population Health, University of Oxford, Oxford, United Kingdom

**Keywords:** healthy lifestyle, cardiovascular diseases, ischemic heart disease, stroke, hypertension, prospective cohort study

## Abstract

**Background:**

Whether lifestyle improvement benefits in reducing cardiovascular diseases (CVD) events extend to hypertensive patients and whether these benefits differ between hypertensive and normotensive individuals is unclear. This study aimed to investigate the associations of an overall healthy lifestyle with the subsequent development of CVD among participants with hypertension and normotension.

**Methods:**

Using data from the Suzhou subcohort of the China Kadoorie Biobank study of 51,929 participants, this study defined five healthy lifestyle factors as nonsmoking or quitting for reasons other than illness; nonexcessive alcohol intake; relatively higher physical activity level; a relatively healthy diet; and having a standard waist circumference and body mass index. We estimated the associations of these lifestyle factors with CVD, ischemic heart disease (IHD) and ischemic stroke (IS).

**Results:**

During a follow-up of 10.1 years, this study documented 6,151 CVD incidence events, 1,304 IHD incidence events, and 2,243 IS incidence events. Compared to those with 0–1 healthy lifestyle factors, HRs for those with 4–5 healthy factors were 0.71 (95% CI: 0.62, 0.81) for CVD, 0.56 (95% CI: 0.42, 0.75) for IHD, and 0.63 (95% CI: 0.51, 0.79) for IS among hypertensive participants. However, we did not observe this association among normotensive participants. Stratified analyses showed that the association between a healthy lifestyle and IHD risk was stronger among younger participants, and the association with IS risk was stronger among hypertensive individuals with lower household incomes.

**Conclusion:**

Adherence to a healthy lifestyle pattern is associated with a lower risk of cardiovascular diseases among hypertensive patients, but this benefit is not as pronounced among normotensive patients.

## Introduction

Cardiovascular diseases (CVD) continue to be the leading cause of death and disability globally (1). Moreover, CVD contribute tremendously to the disease burden in China; more than 40% of deaths are attribute to CVD. Ischemic heart disease (IHD) and ischemic stroke (IS) constitute the largest proportions in CVD deaths (2). Meanwhile, as one of the most important independent risk factors for CVD, hypertension had a prevalence rate of 27.5% among Chinese adults in 2018 (3). A third of CVD deaths among hypertensive patients are caused by high blood pressure (4).

It has been demonstrated that avoiding smoking (5), nonexcessive alcohol consumption (6), engaging in adequate physical activity (7, 8), following a healthy diet (9–11), and maintaining a healthy body shape (12) can prevent many cases of CVD in general populations. A healthy lifestyle including these factors was associated with an approximately 43.2% reduction in IHD incidence and a 39.1% reduction in IS incidence among Chinese adults according to previous study (13). However, there is still insufficient research evidence to confirm whether the control of CVD by lifestyle improvement could be extrapolated to hypertensive patients. In addition, it is also unclear whether these healthy lifestyle habits differ between hypertensive and normotensive individuals.

Therefore, this large prospective cohort study aimed to investigate the associations of healthy lifestyle with incidence of CVD, IHD, and IS among Chinese adults with and without hypertension.

## Methods

### Study population

We used participants’ data from the China Kadoorie Biobank (CKB) study in Wuzhong District of Suzhou city, Jiangsu Province. Detailed descriptions of the CKB cohort have been previously published (14–16). We collected informed consent from all participants who completed questionnaires administered by interviewers and had physical measurements taken. The CKB study was approved by the Ethical Review Committee of the Chinese Center for Disease Control and Prevention (Beijing, China), and the Oxford Tropical Research Ethics Committee (University of Oxford, UK).

Overall, a total of 53,269 participants aged 30–79 years were eligible for inclusion. After excluding individuals who had self-reported previous medical histories of cancer (*n* = 331), stroke (*n* = 466), heart disease (*n* = 574) and outliers of duration of hypertension (*n* = 8), current analysis included 51,921 participants.

### Assessment of lifestyle factors

In the baseline questionnaire, a variety of lifestyle factors were assessed. Questions about cigarette smoking included smoking status (never, former, or current smoker), amount of daily cigarette smoking for current smokers, and the reason for quitting and years since quitting for former smokers. Alcohol consumption included drinking status (never, former, occasionally, monthly, weekly, or daily); drinkers who drank once or more per week were asked how much alcohol they consumed on a typical drinking day over the past year. The information about physical activity included the common type (occupational-, commuting-, domestic-, and leisure time-related domains) and duration of activities in the past year. Based on the metabolic equivalent tasks (METs) for each activity, we calculated the daily level of physical activity by multiplying the MET value for each activity by hours spent on each activity and summing the MET-hours for all activities (17). Dietary data was collected by a qualitative food frequency questionnaire including 12 conventional food groups in China to assess the habitual dietary intake during the past year. The relative validity and reproducibility of qualitative and quantitative food frequency questionnaires (FFQs) have been validated in previous studies (18). Weight, height, and waist circumference (WC) were measured by trained investigator using calibrated instruments. We calculated body mass index (BMI) as weight (kg)/(height (m)^2^).

### Assessment of covariates and hypertension

Baseline questionnaire also collected sociodemographic information (age, sex, marital status, highest education level, household income, and occupation), personal and family medical history, time of sedentary behavior, consumption of preserved vegetable and use of antihypertensive drugs. Participants reporting at least one first-degree relative with stroke or heart attack were defined as having a family history of these diseases. Participants were asked how many hours per week they spent watching TV or reading to calculate the time of sedentary behavior per week. Trained staff members used a UA-779 digital monitor to measure blood pressure at least twice, using the mean of the 2 measurements for analyses. Self-reported diabetes or screen-detected diabetes were considered as diabetes mellitus (19). Screen-detected diabetes was defined as measured nonfasting blood glucose ≥11.1 mmol/L or fasting blood glucose ≥7.0 mmol/L (20). Participants with self-reported diagnosis of hypertension by a registered physician, measured systolic blood pressure ≥ 140 mmHg, measured diastolic blood pressure ≥ 90 mmHg, or self-reported use of antihypertensive medication at baseline were classified as having hypertension (4). Duration of hypertension was calculated as age at baseline minus age at diagnosis of hypertension. If hypertension was ascertained by blood pressure at baseline, the duration of hypertension would be considered as 0 year.

### Definition of healthy lifestyle

Smoking status, alcohol intake, physical activity, diet, and body shape have been proven to be closely related to the risks of CVD. We included these five lifestyle factors to define a healthy lifestyle (21–24). The healthy group regarding smoking status was defined as nonsmokers or individuals who stopped smoking not resulting from illness (25) because there may be a misleadingly elevated risk while including those who quitted smoking due to illness. For alcohol consumption, the healthy group was defined as never drinkers, weekly drinkers, and moderate daily drinkers (i.e., drinking <25 g of pure alcohol for men and < 15 g for women per day) (26). The healthy group for physical activity was defined as those whose physical activity level was above median after taking age- (<50 years, 50–59 years, and ≥ 60 years) and sex-specific into account. For diet, according to the Chinese Dietary Guidelines and previous findings (10, 11, 27), six food items were taken into consideration, including vegetables, fruits, eggs, red meat, grains and fish. We created a diet score according to the following criteria: eating vegetables daily, eating fruits daily, eating eggs ≥4 days every week, eating red meat 1–6 days every week, eating grains weekly, eating fish weekly. A score of 1 for those who met the criteria for each food group, a score of 0 otherwise. The diet score ranged from 0 to 6. The healthy group included participants who scored 4 to 6. For body shape, we took body weight and fat into consideration to reflect energy balance. The healthy group was defined as having a moderate BMI (18.5 ≤ BMI ≤ 27.9 kg/m^2^) and WC (WC < 90 cm for men and < 85 cm for women). The healthy lifestyle score ranged from 0 to 5. To avoid extreme groups with limited cases, we subsequently categorized the lifestyle scores into four groups (0–1, 2, 3, and 4–5).

### Ascertainment of outcomes

Information on CVD incidence cases since baseline recruitment was ascertained from local disease and death registries, the health insurance system, and active follow-up (14). The health insurance system has almost universal coverage and includes detailed descriptions of diagnosis. Street committees conduct annual surveys to supplement the morbidity information of uninsured participants. Trained investigators blinded to baseline information coded all cases with the 10th revision of the International Classification of Diseases (ICD-10). Major cardiovascular events (for stroke, IHD) were reviewed and integrated centrally by cardiovascular specialists from China and the UK.

The primary outcomes were incidences of total cardiovascular diseases (CVD), ischemic heart disease (IHD) and ischemic stroke (IS). Total CVD included all circulatory diseases coded as “I” in ICD-10 (e.g., stroke, any type of heart disease, peripheral vascular disease) and were coded as I00 to I99. IHD and IS were coded as I20 to I25 and I63, which were subdivisions of total CVD.

### Statistical analysis

Participants contributed person-years from enrollment into the study until the diagnosis of CVD, loss to follow-up, or December 31, 2017, whichever came first. A Cox proportional hazards model was used to estimate the hazard ratios (HRs) and 95% confidence intervals (CIs) for the associations of individual and combined lifestyle factors with risks of incidence of total CVD, IHD and IS among participants with or without hypertension. The Cox model was stratified by age at baseline in 5-year intervals.

All lifestyles were included when analyzing individual lifestyle factors. Model 1 was adjusted for sex. Model 2 was additionally adjusted for education level, occupation, marital status, family history of heart attack or stroke, time of sedentary behavior, and usage of antihypertensive drugs. Similarly, we made the same adjustments in the analysis of combined lifestyle factors. We treated the number of healthy lifestyle factors as a continuous variable to analyze the linear trend. Analyses were further stratified by age, sex, education level, household income, occupation, time of sedentary behavior, and usage of antihypertensive drugs for hypertension. The likelihood ratio test including the cross-product term was used to estimate multiplicative interactions. To demonstrate the robustness of our findings, we conducted several sensitivity analyses. First, participants who had diabetes at baseline were excluded. Second, participants whose outcome occurred in the first 2 years of follow-up were excluded. Third, participants whose BMI < 18.5 kg/m^2^ were excluded. Forth, for alcohol consumption, the healthy group was redefined as moderate drinking.

R software (version 4.1.0) was used to perform the statistical analyses. A two-sided *p* < 0.05 was considered statistically significant.

## Results

### Baseline characteristics

Table 1; Supplementary Table S1 show the characteristics of the study participants with or without hypertension (mean age 51.87 years, 58.14% women). Of the 20,194 hypertensive participants, 27.18, 37.84 and 25.31% had 2, 3, and ≥ 4 healthy lifestyle factors, while for the 31,727 normotensive participants, 20.91, 38.77 and 34.62% had 2, 3, and ≥ 4 healthy lifestyle factors. Women were more likely to adhere to a healthy lifestyle. Married participants tended to adhere to fewer healthy lifestyle behaviors.

**Table 1:** Baseline characteristics of participants according to the number of healthy lifestyle factors.

Baseline characteristics	Number of healthy lifestyle factors				*p* value^b^
	0–1	2	3	4–5	
Hypertension					
No. of participants	1,953 (9.67)	5,488 (27.18)	7,642 (37.84)	5,111 (25.31)	
Age, years	53.40 (9.85)	56.47 (9.78)	56.89 (9.82)	56.36 (9.75)	<0.01
Women	23 (1.18)	2,074 (37.79)	4,853 (63.50)	4,293 (84.00)	<0.01
Married	1,849 (94.67)	5,027 (91.60)	6,819 (89.23)	4,548 (88.98)	<0.01
High school and above	258 (13.21)	466 (8.49)	584 (7.64)	452 (8.84)	<0.01
Household income ≥20,000 RMB/year	1,507 (77.16)	3,718 (67.75)	4,981 (65.18)	3,346 (65.47)	<0.01
Family history of heart attack or stroke	491 (25.14)	1,410 (25.69)	2,067 (27.05)	1,294 (25.32)	0.29
Low-risk lifestyle factors^a^					
Nonsmoking	40 (2.05)	2,374 (43.26)	5,653 (73.97)	4,910 (96.07)	<0.01
Nonexcessive alcohol intake	831 (42.55)	4,492 (81.85)	7,410 (96.96)	5,102 (99.82)	<0.01
Being physically active	232 (11.88)	1,443 (26.29)	3,584 (46.90)	4,379 (85.68)	<0.01
Healthy dietary habits	38 (1.95)	341 (6.21)	1,138 (14.89)	1,906 (37.29)	<0.01
Healthy body weight and fat	561 (28.73)	2,326 (42.38)	5,141 (67.27)	4,715 (92.25)	<0.01
Normotension					
No. of participants	1,807 (5.70)	6,634 (20.91)	12,302 (38.77)	10,984 (34.62)	
Age, years	49.44 (9.33)	50.08 (9.55)	49.25 (9.56)	48.13 (9.47)	<0.01
Women	20 (1.11)	1,701 (25.64)	7,534 (61.24)	9,687 (88.19)	<0.01
Married	1,737 (96.13)	6,307 (95.07)	11,587 (94.19)	10,344 (94.17)	<0.01
High school and above	243 (13.45)	788 (11.88)	1,139 (9.26)	1,117 (10.17)	<0.01
Household income ≥20,000 RMB/year	1,459 (80.74)	5,092 (76.76)	9,487 (77.12)	8,697 (79.18)	<0.01
Family history of heart attack or stroke	336 (18.59)	1,154 (17.40)	2,180 (17.72)	1,939 (17.65)	0.67
Low-risk lifestyle factors^a^					
Nonsmoking	28 (1.55)	1,865 (28.11)	8,286 (67.35)	10,519 (95.77)	<0.01
Nonexcessive alcohol intake	733 (40.56)	5,331 (80.36)	12,028 (97.77)	10,961 (99.79)	<0.01
Being physically active	147 (8.14)	1,727 (26.03)	5,278 (42.90)	9,103 (82.88)	<0.01
Healthy dietary habits	31 (1.72)	340 (5.13)	1,435 (11.66)	4,290 (39.06)	<0.01
Healthy body weight and fat	711 (39.35)	4,005 (60.37)	9,879 (80.30)	10,555 (96.09)	<0.01

Data are presented as means (SDs) for continuous variables or *n* (%) for categorical variables. ^a^Healthy lifestyle factors were defined as follows: nonsmoking or having stopped for reasons other than illness; nondaily drinking or daily moderate drinking (drinking <25 g of pure alcohol for men and < 15 g for women per day); engaging in an age- (< 50 years, 50–59 years, and ≥ 60 years) and sex-specific median or higher level of physical activity; diet score ≥4; and having a BMI between 18.5 and 27.9 kg/m^2^ and a WC < 90 cm (men)/85 cm (women). ^b^Continuous variables were compared by one-way analysis of variance. Categorical variables were compared by Pearson’s χ^2^ test between different numbers of healthy lifestyles.

### Associations of individual healthy lifestyle factors with the incidence of cardiovascular diseases

During a median follow-up period of 10.1 years, 6,151 incidence of total CVD cases, 1,304 IHD cases, and 2,243 IS cases were documented. When categorizing the five lifestyle factors into healthy and unhealthy groups (reference group), nonsmoking, being physically active, healthy body weight and fat were each independently associated with a 16, 8, and 10% lower risk of the incidence of total CVD, 18, 20, and 18% lower risk of incident IHD, and 26, 3, and 13% lower risk of incident IS among participants with hypertension, respectively. Those associations were only observed between healthy dietary habits and incident CVD among normotensive participants (Table 2).

**Table 2:** Multivariable-adjusted HRs (95% CIs) for incident major cardiovascular diseases (CVD) according to healthy lifestyle factors.

	Hypertension (*n* = 20,202)			Normotension (*n* = 31,727)		
	Cases/PYs	Model 1	Model 2	Cases/PYs	Model 1	Model 2
Total CVD						
Nonsmoking	2,572/133,556	0.83 (0.75, 0.93)	0.84 (0.75, 0.93)	1,354/225,411	0.87 (0.74, 1.03)	0.87 (0.73, 1.02)
Nonexcessive alcohol intake	3,579/181,386	1.10 (0.99, 1.23)	1.06 (0.95, 1.18)	1,958/313,726	1.11 (0.95, 1.31)	1.10 (0.94, 1.30)
Physically active	1,728/99,070	0.88 (0.82, 0.93)	0.92 (0.85, 0.99)	1,049/175,700	0.91 (0.84, 0.99)	0.97 (0.88, 1.07)
Healthy dietary habits	627/34,930	0.89 (0.82, 0.97)	0.89 (0.81, 0.97)	336/65,796	0.84 (0.74, 0.94)	0.84 (0.74, 0.95)
Healthy body weight and fat	2,512/129,899	0.85 (0.80, 0.91)	0.90 (0.84, 0.96)	1,639/272,008	0.90 (0.81, 1.00)	0.90 (0.82, 1.00)
Ischemic Heart Disease						
Nonsmoking	566/140,577	0.84 (0.67, 1.05)	0.82 (0.66, 1.03)	262/228,943	0.83 (0.58, 1.18)	0.85 (0.59, 1.22)
Nonexcessive alcohol intake	803/190,924	1.33 (1.03, 1.70)	1.25 (0.97, 1.61)	398/318,630	1.85 (1.21, 2.82)	1.85 (1.21, 2.82)
Being physically active	343/104,042	0.75 (0.66, 0.86)	0.80 (0.68, 0.95)	201/178,438	0.88 (0.72, 1.07)	0.92 (0.73, 1.15)
Healthy dietary habits	155/36,540	1.02 (0.85, 1.21)	0.93 (0.77, 1.13)	75/ 66,615	0.97 (0.75, 1.24)	1.03 (0.79, 1.35)
Healthy body weight and fat	534/136,510	0.78 (0.68, 0.89)	0.82 (0.71, 0.94)	315/276,162	0.84 (0.67, 1.05)	0.84 (0.67, 1.05)
Ischemic Stroke						
Nonsmoking	975/139,064	0.73 (0.62, 0.88)	0.74 (0.62, 0.88)	442/228,501	1.03 (0.78, 1.36)	1.05 (0.80, 1.39)
Nonexcessive alcohol intake	1,372/188,928	0.97 (0.81, 1.14)	0.92 (0.78, 1.10)	626/318,008	1.06 (0.81, 1.41)	1.07 (0.81, 1.41)
Being physically active	683/102,747	0.90 (0.81, 1.00)	0.97 (0.86, 1.09)	337/178,105	0.92 (0.79, 1.07)	0.96 (0.80, 1.14)
Healthy dietary habits	232/36,278	0.86 (0.74, 0.98)	0.88 (0.76, 1.02)	103/66,564	0.80 (0.65, 0.99)	0.84 (0.67, 1.04)
Healthy body weight and fat	966/134,935	0.82 (0.74, 0.91)	0.87 (0.79, 0.97)	533/275,553	0.97 (0.81, 1.16)	0.97 (0.81, 1.16)

HRs were calculated in the Cox proportional hazards model. Model 1 was adjusted for sex (men or women); Model 2 was further adjusted for education level (no formal school, primary school, middle school, high school, college, or university or above); marital status (married, widowed, divorced or separated, or never married); household income (<20,000 RMB/Y, 20,000 ~ 34,999 RMB/Y, ≥35,000 RMB/Y); family history of heart attack or stroke (presence, absence, or unknown); consumption of preserved vegetable (never/rarely, monthly, 1–3 days per week, 4–6 days per week, daily); occupation (agriculture/factory/service workers or other); sedentary behavior; and use of antihypertensive drugs (non-use, conversion enzyme inhibitors, β-blockers, diuretics, calcium antagonists and other) and duration of hypertension (for hypertensive population only). All five lifestyle factors were included simultaneously in the same model. Healthy lifestyle factors were defined as follows: nonsmoking or having stopped for reasons other than illness; nondaily drinking or daily moderate drinking (drinking <25 g of pure alcohol for men and < 15 g for women per day); engaging in an age- (<50 years, 50–59 years, and ≥ 60 years) and sex-specific median or higher level of physical activity; diet score ≥4; and having a BMI between 18.5 and 27.9 kg/m^2^ and a WC < 90 cm (men)/85 cm (women).

### Association of a healthy lifestyle pattern with the incidence of cardiovascular diseases

When considering healthy lifestyle factors jointly, compared to those with ≤1 healthy lifestyle scores, the adjusted HRs (95% CIs) of those with 4–5 scores were 0.71 (95% CI: 0.62, 0.81) for the incidence of total CVD, 0.56 (95% CI: 0.42, 0.75) for the incidence of IHD, and 0.63 (95% CI: 0.51, 0.79) for the incidence of IS among hypertensive patients (all *p* for trend <0.01) (Table 3). When evaluated ordinally, participants having a 1-score increment were related to a greater magnitude of total CVD, IHD and IS risk lowering among hypertensive patients than among normotensive patients. However, there were no significant multiplicative interactions between blood pressure and lifestyle factors on CVD incidence (*p* for interaction = 0.18 for total CVD, 0.16 for IHD, 0.06 for IS).

**Table 3:** Multivariable-adjusted HRs (95% CIs) for incident major cardiovascular diseases (CVD) according to lifestyle score category.

Category	Lifestyle score category^b^				*p* for trend	HR (95% CI) per score point	*p* for interaction^c^
	0–1	2	3	4–5			
Hypertension							
Total CVD							0.18
Cases/PYs	385/19,310	1,164/54,960	1,559/77,410	896/53,492			
Model 1	1.00	0.85 (0.76, 0.96)	0.79 (0.70, 0.89)	0.66 (0.58, 0.76)	<0.01	0.89 (0.86, 0.92)	
Model 2^a^	1.00	0.85 (0.76, 0.96)	0.81 (0.72, 0.91)	0.71 (0.62, 0.81)	<0.01	0.91 (0.88, 0.95)	
Ischemic Heart Disease							0.16
Cases/PYs	94/20,265	257/57,890	346/81,647	184/56,022			
Model 1	1.00	0.74 (0.58, 0.94)	0.68 (0.53, 0.87)	0.53 (0.40, 0.70)	<0.01	0.86 (0.80, 0.93)	
Model 2	1.00	0.74 (0.58, 0.95)	0.70 (0.54, 0.90)	0.56 (0.42, 0.75)	<0.01	0.88 (0.81, 0.95)	
Ischemic Stroke							0.06
Cases/PYs	160/20,106	447/57,227	619/80,705	328/55,426			
Model 1	1.00	0.78 (0.64, 0.93)	0.73 (0.61, 0.88)	0.57 (0.47, 0.71)	<0.01	0.86 (0.81, 0.91)	
Model 2	1.00	0.78 (0.65, 0.95)	0.76 (0.63, 0.92)	0.63 (0.51, 0.79)	<0.01	0.88 (0.83, 0.94)	
Normotension							
Total CVD							
Cases/PYs	131/19,002	528/70,474	850/132,814	638/11,9,917			
Model 1	1.00	1.00 (0.82, 1.21)	0.89 (0.74, 1.08)	0.80 (0.65, 0.98)	<0.01	0.91 (0.87, 0.96)	
Model 2	1.00	1.00 (0.82, 1.21)	0.91 (0.75, 1.11)	0.84 (0.68, 1.04)	0.01	0.93 (0.88, 0.98)	
Ischemic Heart Disease							
Cases/PYs	24/19,321	99/71,709	182/134,933	118/121,643			
Model 1	1.00	1.02 (0.65, 1.60)	1.08 (0.69, 1.69)	0.86 (0.54, 1.39)	0.31	0.94 (0.84, 1.06)	
Model 2	1.00	1.05 (0.67, 1.65)	1.16 (0.74, 1.81)	0.97 (0.59, 1.57)	0.73	0.98 (0.87, 1.10)	
Ischemic Stroke							
Cases/PYs	32/19,331	180/71,428	271/134,663	206/121,464			
Model 1	1.00	1.39 (0.95, 2.02)	1.18 (0.81, 1.73)	1.10 (0.74, 1.64)	0.14	0.94 (0.86, 1.02)	
Model 2	1.00	1.39 (0.95, 2.03)	1.22 (0.83, 1.79)	1.17 (0.78, 1.76)	0.39	0.96 (0.87, 1.05)	

^a^HRs were calculated in the Cox proportional hazards model. Model 1 was adjusted for sex (men or women); Model 2 was further adjusted for education level (no formal school, primary school, middle school, high school, college, or university or above); marital status (married, widowed, divorced or separated, or never married); household income (<20,000 RMB/Y, 20,000 ~ 34,999 RMB/Y, ≥35,000 RMB/Y); family history of heart attack and stroke (presence, absence, or unknown); consumption of preserved vegetable (never/rarely, monthly, 1–3 days per week, 4–6 days per week, daily); occupation (agriculture/factory/service workers or other); sedentary behavior; and use of antihypertensive drugs (non-use, conversion enzyme inhibitors, β-blockers, diuretics, calcium antagonists and other) and duration of hypertension (for hypertensive population only). All five lifestyle factors were included simultaneously in the same model. ^b^Healthy lifestyle factors were defined as nonsmoking or having stopped for reasons other than illness; nondaily drinking or daily moderate drinking (drinking <25 g of pure alcohol for men and < 15 g for women per day); engaging in an age- (<50 years, 50–59 years, and ≥ 60 years) and sex-specific median or higher level of physical activity; diet score ≥4; and having a BMI between 18.5 and 27.9 kg/m^2^ and a WC < 90 cm (men)/85 cm (women). ^c^The *p* for interaction was calculated using multiplicative interaction terms and the likelihood ratio test for the interaction between normotension and hypertension.

### Stratified analyses

**Figure 1:**
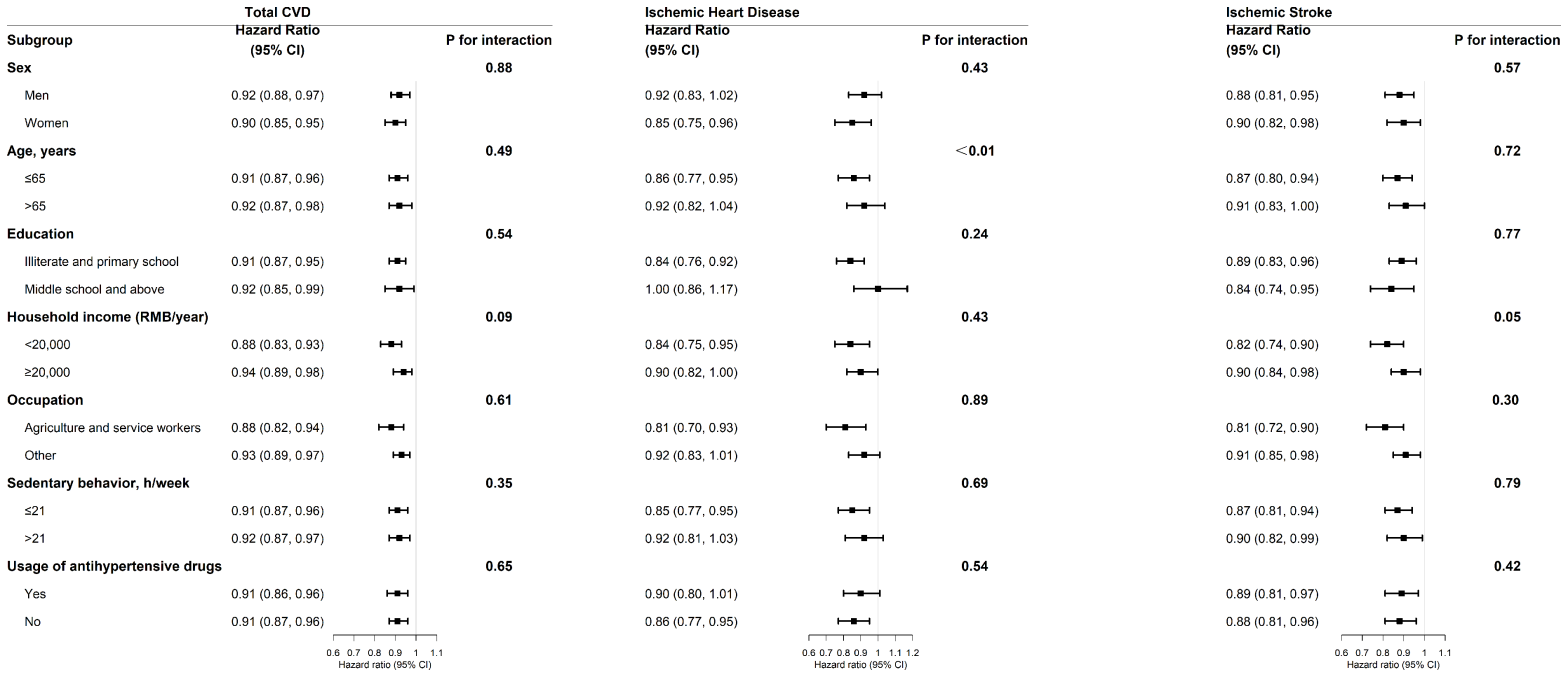
Stratified analysis of the association of incident major cardiovascular diseases (CVD) with each 1-unit increment in healthy lifestyle factors in the hypertensive population. This multivariable model was adjusted for sex, education level, marital status, household income, family history of heart attack or stroke, consumption of preserved vegetable, occupation, sedentary behavior, usage of antihypertensive drugs, and duration of hypertension at baseline. The *p* for interaction was calculated using multiplicative interaction terms and the likelihood ratio test.

When stratified by age, sex, education level, household income, occupation, duration of sedentary behavior, and antihypertensive drug use, the analyses yielded consistent results (Figure 1). For participants with hypertension, adults younger than 65 years had a stronger inverse association between healthy lifestyle scores and IHD risk (*p* for interaction <0.01), and adults with annual household incomes less than 20,000 RMB/year had a stronger inverse association between healthy lifestyle scores and IS risk as well (*p* for interaction = 0.05).

### Sensitivity analyses

Several sensitivity analyses were performed by excluding participants who had diabetes at baseline (Supplementary Table S2), excluding participants whose disease outcomes occurred in the first 2 years of follow-up (Supplementary Table S3), excluding participants whose BMI < 18.5 kg/m^2^ (Supplementary Table S4), and only considering moderate drinking as healthy (Supplementary Table S5). The risk estimates did not have materially changes among sensitivity analyses.

## Discussion

### Principal findings

This large, prospective cohort study of Chinese adults examined the associations of healthy lifestyle scores (i.e., nonsmoking, nonexcessive alcohol intake, being physically active, having a relatively healthy dietary habit, healthy body weight and fat) with the incidence of total CVD, IHD, and IS. Compared with 0 or 1 ideal lifestyle factors, hypertensive patients having a score of 4 or 5 showed a 29, 44, and 37% reduction in the risk of total CVD, IHD, and IS, which was higher than that of normotensive individuals.

### Comparison with other studies

Our findings in hypertensive patients are consistent with previous studies in the general population (13, 23, 28–31). In Nurses’ Health Study of 15 to 20 years follow-up data, the relative risk (RR) for the healthy lifestyle factors including nonsmoking, daily moderate alcohol consumption, moderate-to-vigorous physical activity, a healthy diet, and BMI under 25 kg/m^2^ was 0.25 (95% CI: 0.14, 0.44) for total CVD incidence, 0.17 (95% CI: 0.07, 0.41) for coronary heart disease (CHD) incidence (28), and 0.19 (95% CI: 0.09, 0.40) for IS incidence (29). In Swedish cohorts, a healthy pattern combination of a healthy diet, being physically active, nonsmoking, moderate daily drinking was associated with a population attributable risk of 79% (95% CI: 34, 93%) in myocardial infarction (MI) events among men (23) and a 92% (95% CI: 72, 98%) in MI events among women (30). Lv et al. combined five healthy lifestyles (normal BMI and waist-to-hip ratio, participation in physical exercise, a diet rich in vegetables and fruits and limited in red meat, nonsmoking, and moderate alcohol consumption) to quantify their impacts on IHD and IS incidence in a Chinese population (13). The HR for having 5 to 6 healthy lifestyle factors was 0.50 (95% CI: 0.41, 0.60) for IHD incidence and 0.50 (95% CI: 0.40, 0.64) for IS incidence during the 7.2-year follow-up. However, this protective effect was reduced in the normotensive population in this study. Generally, although many prospective studies have demonstrated the significance of lifestyle interventions for the prevention of CVD, they might have missed patients who already had hypertension. Meanwhile, because adherence to a healthy lifestyle can also reduce the risk of hypertension (32, 33), people without hypertension are recommended to follow a healthy lifestyle as well. Furthermore, due to the potential mediating effect of lipid profile on lifestyle and CVD (34), lipid was not included as a confounder in models, which was consistent with other studies (13, 28–30).

In stratified analyses, the association between healthy lifestyle and IHD risk was stronger among younger participants, and the association with IS risk was stronger among adults with lower household incomes, which was consistent with previous studies (35). These results indicated that people could obtain larger benefits if they adopted healthy lifestyles at an early age or have a lower socioeconomic status. The possible reason may be that individuals of different ages and socioeconomic statuses perceive and choose healthy lifestyles differently, such as people who may choose not to smoke or drink because of financial constraints.

Previous studies have found that compared to people with moderate alcohol consumption, nondrinkers have an increased risk of CVD (13, 36). Nevertheless, compared to nondrinking, moderate drinking can increase the risk of cancer and injury (37, 38). Therefore, regarding overall human health, our study considered nondrinking as a healthy lifestyle. By classifying both nondrinking and moderate drinking participants into low-risk groups, we found that nonexcessive drinking had no independent protective effect on CVD. Meanwhile, the association between healthy lifestyle and CVD slightly changed after only considering moderate drinking as healthy. The reason may be that genetic variants involved in alcohol metabolism (such as rs671 variant, which is common in east Asian populations and can slow the decomposition of acetaldehyde to causes cardiovascular damage) were different in the two groups (39).

### Public health impact

For the primary prevention of CVD, this study’s healthy lifestyle pattern provides a positive framework. Our findings contribute valuable information to the prevention of CVD by five modifiable lifestyle factors in hypertensive populations. However, in this study, only less than one-third participants adopted 4 or 5 healthy lifestyles. From a public health perspective, individuals, especially hypertensive patients, could refer to our findings to better understand the significance of CVD prevention and develop healthy lifestyle habits in reference to our findings.

### Strengths and limitations

The strengths of this study included a prospective study design, a relatively large sample size of population, controlling for potential confounding factors, and the use of measured anthropometric information to provide more accurate estimates of blood pressure, BMI and WC (14). Meanwhile, the present study has several limitations. First, lifestyle behaviors were self-reported, which may lead to some misclassification. However, there is no evidence that this type of exposure misclassification is differentially associated with CVD. Second, we created a healthy lifestyle score by using baseline lifestyle information, there is no measurement on the persistence of lifestyles during the follow-up. However, the re-survey conducted during the follow-up showed that there was good agreement between the baseline and re-survey for lifestyle variables (14). Third, confounding such as genetic susceptibility, detailed medication use, or salt and sugar-sweetened beverage intake could not be entirely ruled out. Unmeasured or unknown factors could still cause residual confounding. Forth, some individuals who self-reported taking blood pressure medications at baseline may have met their blood pressure goals at follow-up, which may weaken the difference of protective effects of healthy lifestyles between hypertensive and normotensive population. In addition, information on adherence and persistence to antihypertensive drugs in hypertensive participants could not be confirmed during the follow-up. However, this study calculated the correlation coefficient between healthy lifestyle scores and use of blood pressure medications, and the correlation coefficient is 0.054, indicating that there was little association between taking blood pressure medications and healthy lifestyle scores at baseline (Supplementary Material S5). Finally, this study was observational, and further RCTs are needed to confirm the causal nature of the associations.

## Conclusion

This prospective cohort study of Chinese adults provided evidence that adopting a healthy lifestyle pattern, including abstinence from or cessation of smoking, nondaily drinking or daily moderate drinking, adequate physical activity, adherence to a healthy diet, and having a standard BMI and WC, is related to a significantly lower risk of the incidence of total CVD, IHD, and IS among hypertensive participants, but this association is not as pronounced among normotensive individuals.

## Data availability statement

The datasets presented in this article are not readily available because the data that support the findings of this study are available from the Department of the China Kadoorie Biobank, but restrictions apply to the availability of these data, which were used under license for the current study and are not publicly available. Data are, however, available from the authors upon reasonable request and with the permission of the Department of the China Kadoorie Biobank. Requests to access the datasets should be directed to https://www.ckbiobank.org/site.

## Ethics statement

The studies involving human participants were reviewed and approved by Ethical Review Committee of the Chinese Center for Disease Control and Prevention (Beijing, China) and the Oxford Tropical Research Ethics Committee, University of Oxford (UK). The patients/participants provided their written informed consent to participate in this study.

## Author contributions

JS and HG designed the study. HG performed the data analyses and drafted the manuscript. JS revised the data analyses. JS, LC, XF, JZ, MW, and RT critically revised the manuscript for important intellectual content. YL, YH, JJ, YG, JL, PP, and ZC edited and proofread the manuscript. All authors read and approved the final manuscript.

## Funding

This work was supported by grants from the National Natural Science Foundation of China (82192900, 81390540, and 91846303), grants from the National Key Research and Development Program of China (2016YFC0900500), grants from the Kadoorie Charitable Foundation in Hong Kong and grants from the Wellcome Trust (088158/Z/09/Z, 104085/Z/14/Z) in the UK.

## Conflict of interest

The authors declare that the research was conducted in the absence of any commercial or financial relationships that could be construed as a potential conflict of interest.

## Publisher’s note

All claims expressed in this article are solely those of the authors and do not necessarily represent those of their affiliated organizations, or those of the publisher, the editors and the reviewers. Any product that may be evaluated in this article, or claim that may be made by its manufacturer, is not guaranteed or endorsed by the publisher.
