# Supplementary material for: Association of healthy lifestyle with incident cardiovascular diseases among hypertensive and normotensive Chinese adults
Source: Front Cardiovasc Med. 2023 Mar 2;10:1046943. doi: 10.3389/fcvm.2023.1046943 (PMC10017485; doi:10.3389/fcvm.2023.1046943)
Supplement: Supplementary file 1 [file Table_1.docx]

Supplementary Material

**Supplementary Material S1. Food frequency questionnaire used in the CKB study at baseline**

**During the past 12 months, about how often did you eat the following foods?**

|  | Daily | 4-6 days/week | 1-3 days/week | Monthly | Never/rarely |
| --- | --- | --- | --- | --- | --- |
| Rice | □ | □ | □ | □ | □ |
| Wheat | □ | □ | □ | □ | □ |
| Other staple food (corn, millet, etc.) | □ | □ | □ | □ | □ |
| Meat | □ | □ | □ | □ | □ |
| Poultry | □ | □ | □ | □ | □ |
| Fish/sea food | □ | □ | □ | □ | □ |
| Fresh eggs | □ | □ | □ | □ | □ |
| Fresh vegetables | □ | □ | □ | □ | □ |
| Soybean products | □ | □ | □ | □ | □ |
| Preserved vegetables | □ | □ | □ | □ | □ |
| Fresh fruit | □ | □ | □ | □ | □ |
| Dairy products (milk, yogurt) | □ | □ | □ | □ | □ |

**Supplementary Material S2. Validation of food frequency questionnaire**

To evaluate the reproducibility and relative validity of baseline FFQ, during 2015-2016, 432 participants were selected from four survey sites of the CKB study. The validity analysis of baseline FFQ used 24-hour dietary recalls as a reference, and the reproducibility was verified by twice administrations of FFQ. In both evaluations, weighted kappa coefficients exceeded 0.60 for all food groups except fresh vegetable. The low kappa of fresh vegetable might be due to its high proportion of agreements on daily intake, instead, the percentage of correct classification was over 89% and the percentage of extreme classification was below 1% for both validity and reproducibility. Therefore, validation study indicated that the FFQ estimated the frequency of dietary intakes with reasonably good validity and reproducibility.

**Supplementary Material S3. Physical activity types, MET values, codes and intensity categories**

| Activity type | Intensity | MET | Codes* |
| --- | --- | --- | --- |
| Heavy manual work | Vigorous | 6.5 | 11477 |
| Manual work | Moderate | 4.5 | 11476 |
| Standing work | Moderate | 3.8 | Mean of 11610 and 11630 |
| Sedentary work | Low | 1.8 | Mean of 11580, 11585, and 11590 |
| Manual work in the farming season | Vigorous | 6.3 | Mean of 11145 and 11146 |
| Semi-mechanized work in the farming season | Moderate | 3.4 | Mean of 11146 and 11147 |
| Fully mechanized work in the farming season | Low | 2.4 | Mean of 11147 and 11170 |
| Work outside the farming season | Low | 2.0 | 11147 |
| Walking | Moderate | 4.0 | 17270 |
| Bicycle | Vigorous | 6.8 | 1011 |
| Motorbike | Moderate | 3.5 | 16030 |
| Private or public transportation (such as bus, car, underground, and ferry) | Low | 1.7 | Mean of 16010, 16015, and 16016 |
| Household activity | Low | 2.8 | Mean of 05030†, 05040†, 05035, 05055, 05070, 05090†, 05092†, 05184, 05197, and 05200 |
| Tai-Chi/qigong/leisure walking | Moderate | 3.3 | Mean of 15670 and 17160 |
| Jogging/aerobic exercise | Vigorous | 7.4 | Mean of 03015, 12020, and 12150 |
| Ball games | Moderate | 5.5 | Mean of 15020†, 15030†, 15055, 15080, 15090, 15255, 15605†, 15610†, 15652, 15660, 15675, 15710†, and 15711† |
| Brisk walking/gymnastics/folk dancing | Moderate | 4.2 | Mean of 03025, 15300, and 17200 |
| Swimming | Vigorous | 7.2 | Mean of 18230, 18240, and 18310 |
| Other exercise, e.g. mountain walking, home exercise and rope jumping | Moderate | 5.9 | Mean of 02010, 02064, 04001, 04100, 15110†, 15120†, 15200, 15240, 15310, 15425†, 15430†, 15537, 15550‡, 15551‡, 15552‡, 15580, 15590, 15730, 15732‡, 15733‡, 15734‡, and 19030 |

MET, metabolic equivalent of task.

*Based on the 2011 Compendium of Physical Activities: a second update of codes and MET values. Ainsworth BE, et al. Medicine and Science in Sports and Exercise, 2011;43(8):1575-1581.

†Assigned 1/2 weight in calculating the mean MET value because the connecting two items represent one type of activity.

‡Assigned 1/3 weight in calculating the mean MET value because the connecting three items represent one type of activity.

**Supplementary Material S4. Description of the components of the healthy lifestyle factors**

| Healthy Lifestyle Factor | Description | Score |
| --- | --- | --- |
| Smoking Status | Nonsmokers or individuals who stopped smoking not resulting from illness | 1 |
|  | Smokers or individuals who stopped smoking resulting from illness | 0 |
| Alcohol Intake | Never drinkers, weekly drinkers, and moderate daily drinkers (drinking < 25 g of pure alcohol for men and < 15 g for women per day) | 1 |
|  | Excessive daily drinkers | 0 |
| Physical Activity | Physical activity level above median after taking age- and sex-specific into account based on METs | 1 |
|  | Physical activity level below median after taking age- and sex-specific into account based on METs | 0 |
| Diet^a^ | Diet score ≥ 4 | 1 |
|  | Diet score < 4 | 0 |
| Body Shape | 18.5 ≤ BMI ≤ 27.9 kg/m^2^, and  WC < 90 cm for men and < 85 cm for women | 1 |
|  | BMI <18.5, or BMI > 27.9 kg/m^2^, or  WC ≥ 90 cm for men and ≥ 85 cm for women | 0 |
| Healthy Lifestyle Score | Sum of every lifestyle’ score above | 0-5 |

BMI: Body Mass Index; WC: Waist circumference; Mets: Metabolic equivalent tasks

^a^Diet included vegetables, fruits, eggs, red meat, grains and fish. A diet score was created according to the following criteria: eating vegetables daily, eating fruits daily, eating eggs ≥4 days every week, eating red meat 1-6 days every week, eating grains weekly, eating fish weekly. A score of 1 for those who meet the criteria for each food group, a score of 0 otherwise. The diet score ranged from 0 to 6.

**Supplementary Material S5. The correlation coefficient between healthy lifestyle and use of antihypertensive medications**

|  | Use of antihypertensive medications | Healthy lifestyle scores | Nonsmoking | Nonexcessive alcohol intake | Being physically active | Healthy dietary habits | Healthy body weight and fat |
| --- | --- | --- | --- | --- | --- | --- | --- |
| Use of antihypertensive medications | 1.000 | 0.054 | 0.066 | 0.098 | 0.072 | 0.072 | 0.125 |
| Healthy lifestyle scores | 0.054 | 1.000 | 0.505 | 0.459 | 0.444 | 0.319 | 0.403 |
| Nonsmoking | 0.066 | 0.505 | 1.000 | 0.352 | 0.017 | 0.049 | 0.069 |
| Nonexcessive alcohol intake | 0.098 | 0.459 | 0.352 | 1.000 | 0.049 | 0.049 | 0.063 |
| Being physically active | 0.072 | 0.444 | 0.017 | 0.049 | 1.000 | 0.056 | 0.071 |
| Healthy dietary habits | 0.051 | 0.319 | 0.046 | 0.049 | 0.056 | 1.000 | 0.052 |
| Healthy body weight and fat | 0.125 | 0.403 | 0.069 | 0.063 | 0.071 | 0.052 | 1.000 |

**Table S1. Baseline characteristics of participants with and without hypertension**

| Baseline characteristics | All participants (n=51,921) | Hypertensive participants (n=20,194) |  | Normotensive participants (n=31,727) | *P* value^b^ |
| --- | --- | --- | --- | --- | --- |
|  |  |  |  |  |  |
| Age, years | 51.87 (10.28) | 56.30 (9.84) |  | 49.05 (9.54) | < 0.01 |
| Women | 30,185 (58.14) | 11,243 (55.67) |  | 18,942 (59.70) | < 0.01 |
| Married | 48,218 (92.87) | 18,243 (90.34) |  | 29,975 (94.48) | < 0.01 |
| Education level |  |  |  |  | < 0.01 |
| No formal school | 15,453 (29.76) | 7,777 (38.51) |  | 7,676 (24.19) |  |
| Primary school | 16,787 (32.33) | 6,387 (31.63) |  | 10,400 (32.78) |  |
| Middle school | 14,634 (28.19) | 4,270 (21.14) |  | 10,364 (32.67) |  |
| High school | 4,072 (7.84) | 1,364 (6.75) |  | 2,708 (8.54) |  |
| College/University and above | 975 (1.88) | 396 (1.96) |  | 579 (1.82) |  |
| Household income |  |  |  |  | < 0.01 |
| ＜20,000 RMB/year | 13,634 (26.26) | 6,642 (32.89) |  | 6,992 (22.04) |  |
| 20,000~34,999 RMB/year | 16,443 (31.67) | 5,786 (28.65) |  | 10,657 (33.59) |  |
| ≥35,000 RMB/year | 21,844 (42.07) | 7,766 (38.46) |  | 14,078 (44.37) |  |
| Agriculture/Factory/Service workers | 26,645 (51.32) | 8,959 (44.36) |  | 17,686 (55.74) | < 0.01 |
| Sedentary behavior, h/week | 21.21 (14.22) | 21.47 (15.16) |  | 21.04 (13.58) | 0.57 |
| Use of antihypertensive drugs | 8,134 (15.67) | 8,134 (40.28) |  | NA |  |
| Family history of heart attack or stroke | 10,871 (20.94) | 5,262 (26.06) |  | 5,609 (17.68) | < 0.01 |
| Systolic blood pressure, mmHg | 132.58 (20.25) | 150.86 (18.15) |  | 120.94 (10.63) | < 0.01 |
| Diastolic blood pressure, mmHg | 78.90 (10.33) | 86.31 (9.94) |  | 74.18 (7.38) | < 0.01 |
| No. of healthy lifestyle factors^a^ |  |  |  |  | < 0.01 |
| 0-1 | 3,760 (7.24) | 1,953 (9.67) |  | 1,807 (5.70) |  |
| 2 | 12,122 (23.35) | 5,488 (27.18) |  | 6,634 (20.91) |  |
| 3 | 19,944 (38.41) | 7,642 (37.84) |  | 12,302 (38.77) |  |
| 4-5 | 16,095 (31.00) | 5,111 (25.31) |  | 10,984 (34.62) |  |

Data are presented as means (SDs) for continuous variables or n (%) for categorical variables.

^a^Healthy lifestyle factors were defined as follows: nonsmoking or having stopped for reasons other than illness; nondaily drinking or daily moderate drinking (drinking <25 g of pure alcohol for men and <15 g for women per day); engaging in an age- (< 50 years, 50–59 years, and ≥ 60 years) and sex-specific median or higher level of physical activity; diet score≥4; and having a BMI between 18.5 and 27.9 kg/m^2^ and a WC < 90 cm (men)/85 cm (women).

^b^Continuous variables were compared by one-way analysis of variance, and categorical variables were compared by Pearson’s χ^2^ test between hypertensive and normotensive participants.

| **Table S2. Multivariable-adjusted HRs (95% CIs) for incident major cardiovascular diseases (CVDs) according to lifestyle score category when excluding the participants who had diabetes at baseline** | | | | | | |
| --- | --- | --- | --- | --- | --- | --- |
| Category | Lifestyle score category | | | | *P* for trend | HR (95% CI) per score point |
|  | 0-1 | 2 | 3 | 4-5 |  |  |
| **Hypertension** |  |  |  |  |  |  |
| Total CVD |  |  |  |  |  |  |
| Cases/PYs | 338/17,647 | 1000/49,934 | 1,399/71,390 | 800/50,245 |  |  |
| Model 1 | 1.00 | 0.83 (0.73, 0.94) | 0.78 (0.68, 0.89) | 0.64 (0.55, 0.74) | <0.01 | 0.88 (0.85, 0.92) |
| Model 2 | 1.00 | 0.83 (0.73, 0.94) | 0.79 (0.70, 0.91) | 0.67 (0.58, 0.78) | <0.01 | 0.90 (0.87, 0.94) |
| Ischemic Heart Disease |  |  |  |  |  |  |
| Cases/PYs | 85/18,470 | 221/52,481 | 313/75,097 | 157/52,490 |  |  |
| Model 1 | 1.00 | 0.70 (0.54, 0.91) | 0.66 (0.51, 0.86) | 0.48 (0.36, 0.65) | <0.01 | 0.85 (0.78, 0.92) |
| Model 2 | 1.00 | 0.70 (0.54, 0.91) | 0.68 (0.52, 0.88) | 0.50 (0.37, 0.68) | <0.01 | 0.86 (0.79, 0.93) |
| Ischemic Stroke |  |  |  |  |  |  |
| Cases/PYs | 136/18,359 | 371/51,926 | 537/74,409 | 283/51,980 |  |  |
| Model 1 | 1.00 | 0.75 (0.61, 0.92) | 0.72 (0.59, 0.89) | 0.56 (0.44, 0.70) | <0.01 | 0.85 (0.80, 0.91) |
| Model 2 | 1.00 | 0.75 (0.61, 0.92) | 0.74 (0.60, 0.91) | 0.59 (0.47, 0.76) | <0.01 | 0.87 (0.82, 0.93) |
| **Normotension** |  |  |  |  |  |  |
| Total CVD |  |  |  |  |  |  |
| Cases/PYs | 119/18,336 | 491/67,747 | 802/128,990 | 608/117,200 |  |  |
| Model 1 | 1.00 | 1.02 (0.84, 1.25) | 0.92 (0.75, 1.13) | 0.83 (0.67, 1.02) | <0.01 | 0.92 (0.87, 0.97) |
| Model 2 | 1.00 | 1.02 (0.84, 1.25) | 0.94 (0.77, 1.15) | 0.87 (0.70, 1.08) | 0.01 | 0.93 (0.88, 0.99) |
| Ischemic Heart Disease |  |  |  |  |  |  |
| Cases/PYs | 22/18,632 | 90/68,917 | 170/130,958 | 114/118,854 |  |  |
| Model 1 | 1.00 | 1.02 (0.64, 1.63) | 1.12 (0.71, 1.78) | 0.93 (0.57, 1.52) | 0.55 | 0.96 (0.86, 1.09) |
| Model 2 | 1.00 | 1.06 (0.66, 1.69) | 1.20 (0.75, 1.91) | 1.05 (0.63, 1.74) | 0.95 | 1.00 (0.89, 1.14) |
| Ischemic Stroke |  |  |  |  |  |  |
| Cases/PYs | 24/18,658 | 168/68,641 | 251/130,735 | 192/118,705 |  |  |
| Model 1 | 1.00 | 1.73 (1.13, 2.66) | 1.46 (0.95, 2.25) | 1.35 (0.86, 2.12) | 0.27 | 0.95 (0.86, 1.04) |
| Model 2 | 1.00 | 1.74 (1.13, 2.68) | 1.51 (0.98, 2.33) | 1.44 (0.91, 2.28) | 0.58 | 0.97 (0.88, 1.07) |

HRs were calculated in the Cox proportional hazards model. Model 1 was adjusted for sex; Model 2 was further adjusted for education level, marital status, household income, family history of heart attack or stroke, consumption of preserved vegetable, occupation, sedentary behavior, and use of antihypertensive drugs and duration of hypertension (for hypertensive population only).

| **Table S3. Multivariable-adjusted HRs (95% CIs) for incident major cardiovascular diseases (CVDs) according to lifestyle score category when excluding the participants whose disease outcomes occurred in the first 2 years of follow-up** | | | | | | |
| --- | --- | --- | --- | --- | --- | --- |
| Category | Lifestyle score category | | | | *P* for trend | HR (95% CI) per score point |
|  | 0-1 | 2 | 3 | 4-5 |  |  |
| **Hypertension** |  |  |  |  |  |  |
| Total CVD |  |  |  |  |  |  |
| Cases/PYs | 347/19,267 | 1,088/54,870 | 1,457/77,290 | 853/53,445 |  |  |
| Model 1 | 1.00 | 0.88 (0.78, 1.00) | 0.80 (0.71, 0.91) | 0.69 (0.60, 0.79) | <0.01 | 0.89 (0.86, 0.93) |
| Model 2 | 1.00 | 0.88 (0.78, 1.00) | 0.83 (0.73, 0.94) | 0.73 (0.64, 0.85) | <0.01 | 0.92 (0.88, 0.95) |
| Ischemic Heart Disease |  |  |  |  |  |  |
| Cases/PYs | 86/20,258 | 241/57,871 | 326/81,621 | 176/56,016 |  |  |
| Model 1 | 1.00 | 0.75 (0.58, 0.97) | 0.69 (0.53, 0.89) | 0.54 (0.41, 0.72) | <0.01 | 0.86 (0.80, 0.93) |
| Model 2 | 1.00 | 0.75 (0.58, 0.97) | 0.70 (0.54, 0.92) | 0.57 (0.42, 0.76) | <0.01 | 0.88 (0.81, 0.95) |
| Ischemic Stroke |  |  |  |  |  |  |
| Cases/PYs | 151/20,095 | 420/57,191 | 582/80,663 | 311/55,405 |  |  |
| Model 1 | 1.00 | 0.77 (0.64, 0.94) | 0.73 (0.60, 0.89) | 0.58 (0.47, 0.72) | <0.01 | 0.86 (0.81, 0.91) |
| Model 2 | 1.00 | 0.78 (0.64, 0.94) | 0.76 (0.62, 0.93) | 0.64 (0.51, 0.80) | <0.01 | 0.89 (0.83, 0.95) |
| **Normotension** |  |  |  |  |  |  |
| Total CVD |  |  |  |  |  |  |
| Cases/PYs | 118/18,987 | 507/70,454 | 819/132,775 | 614/119,886 |  |  |
| Model 1 | 1.00 | 1.06 (0.87, 1.30) | 0.95 (0.78, 1.17) | 0.85 (0.69, 1.05) | <0.01 | 0.92 (0.87, 0.97) |
| Model 2 | 1.00 | 1.07 (0.87, 1.31) | 0.97 (0.79, 1.19) | 0.89 (0.72, 1.11) | 0.01 | 0.93 (0.88, 0.99) |
| Ischemic Heart Disease |  |  |  |  |  |  |
| Cases/PYs | 20/19,316 | 94/71,704 | 172/134,920 | 108/121,630 |  |  |
| Model 1 | 1.00 | 1.16 (0.71, 1.88) | 1.22 (0.75, 1.97) | 0.94 (0.56, 1.57) | 0.35 | 0.94 (0.84, 1.06) |
| Model 2 | 1.00 | 1.19 (0.73, 1.94) | 1.29 (0.79, 2.09) | 1.03 (0.61, 1.74) | 0.70 | 0.98 (0.86, 1.11) |
| Ischemic Stroke |  |  |  |  |  |  |
| Cases/PYs | 29/19,329 | 177/71,425 | 266/134,659 | 202/121,460 |  |  |
| Model 1 | 1.00 | 1.50 (1.01, 2.23) | 1.28 (0.86, 1.90) | 1.18 (0.78, 1.79) | 0.17 | 0.94 (0.86, 1.03) |
| Model 2 | 1.00 | 1.51 (1.01, 2.24) | 1.32 (0.88, 1.97) | 1.26 (0.82, 1.92) | 0.42 | 0.96 (0.87, 1.06) |

HRs were calculated in the Cox proportional hazards model. Model 1 was adjusted for sex; Model 2 was further adjusted for education level, marital status, household income, family history of heart attack or stroke, consumption of preserved vegetable, occupation, sedentary behavior, and use of antihypertensive drugs and duration of hypertension (for hypertensive population only).

| **Table S4. Multivariable-adjusted HRs (95% CIs) for incident major cardiovascular diseases (CVDs) according to lifestyle score category when excluding the participants whose BMI was < 18.5 kg/m^2^** | | | | | | |
| --- | --- | --- | --- | --- | --- | --- |
| Category | Lifestyle score category | | | | *P* for trend | HR (95% CI) per score point |
|  | 0-1 | 2 | 3 | 4-5 |  |  |
| **Hypertension** |  |  |  |  |  |  |
| Total CVD |  |  |  |  |  |  |
| Cases/PYs | 362/18,846 | 1,124/53,775 | 1,528/76,409 | 894/53,431 |  |  |
| Model 1 | 1.00 | 0.86 (0.76, 0.97) | 0.79 (0.70, 0.89) | 0.67 (0.58, 0.76) | <0.01 | 0.89 (0.86, 0.92) |
| Model 2 | 1.00 | 0.87 (0.77, 0.98) | 0.82 (0.72, 0.93) | 0.72 (0.63, 0.83) | <0.01 | 0.91 (0.88, 0.95) |
| Ischemic Heart Disease |  |  |  |  |  |  |
| Cases/PYs | 90/19,765 | 249/56,627 | 337/80,571 | 184/55,954 |  |  |
| Model 1 | 1.00 | 0.73 (0.57, 0.94) | 0.65 (0.51, 0.84) | 0.52 (0.39, 0.68) | <0.01 | 0.85 (0.79, 0.92) |
| Model 2 | 1.00 | 0.74 (0.57, 0.95) | 0.68 (0.53, 0.88) | 0.55 (0.41, 0.74) | <0.01 | 0.87 (0.81, 0.95) |
| Ischemic Stroke |  |  |  |  |  |  |
| Cases/PYs | 154/19,603 | 431/55,990 | 613/79,618 | 328/55,358 |  |  |
| Model 1 | 1.00 | 0.76 (0.63, 0.92) | 0.72 (0.59, 0.87) | 0.56 (0.45, 0.69) | <0.01 | 0.85 (0.80, 0.90) |
| Model 2 | 1.00 | 0.76 (0.63, 0.92) | 0.75 (0.62, 0.91) | 0.62 (0.50, 0.78) | <0.01 | 0.88 (0.83, 0.94) |
| **Normotension** |  |  |  |  |  |  |
| Total CVD |  |  |  |  |  |  |
| Cases/PYs | 107/17,457 | 480/66,092 | 823/128,894 | 633/119,258 |  |  |
| Model 1 | 1.00 | 1.05 (0.85, 1.30) | 0.94 (0.76, 1.16) | 0.83 (0.66, 1.04) | <0.01 | 0.91 (0.86, 0.96) |
| Model 2 | 1.00 | 1.06 (0.86, 1.31) | 0.96 (0.78, 1.19) | 0.88 (0.70, 1.11) | 0.01 | 0.93 (0.88, 0.98) |
| Ischemic Heart Disease |  |  |  |  |  |  |
| Cases/PYs | 21/17,736 | 86/67,255 | 179/130,936 | 116/120,979 |  |  |
| Model 1 | 1.00 | 0.95 (0.59, 1.53) | 1.08 (0.67, 1.72) | 0.83 (0.50, 1.37) | 0.36 | 0.95 (0.84, 1.07) |
| Model 2 | 1.00 | 0.98 (0.61, 1.59) | 1.15 (0.72, 1.85) | 0.94 (0.56, 1.57) | 0.81 | 0.99 (0.87, 1.12) |
| Ischemic Stroke |  |  |  |  |  |  |
| Cases/PYs | 29/17,742 | 165/66,971 | 261/130,698 | 204/120,795 |  |  |
| Model 1 | 1.00 | 1.30 (0.87, 1.94) | 1.07 (0.72, 1.60) | 0.98 (0.64, 1.48) | 0.04 | 0.91 (0.83, 1.00) |
| Model 2 | 1.00 | 1.31 (0.88, 1.95) | 1.11 (0.74, 1.66) | 1.05 (0.69, 1.61) | 0.16 | 0.93 (0.84, 1.03) |

HRs were calculated in the Cox proportional hazards model. Model 1 was adjusted for sex; Model 2 was further adjusted for education level, marital status, household income, family history of heart attack or stroke, consumption of preserved vegetable, occupation, sedentary behavior, and use of antihypertensive drugs and duration of hypertension (for hypertensive population only).

| **Table S5. Multivariable-adjusted HRs (95% CIs) for incident major cardiovascular diseases (CVDs) according to lifestyle score category when only considering moderate drinking as a healthy lifestyle** | | | | | | |
| --- | --- | --- | --- | --- | --- | --- |
| Category | Lifestyle score category | | | | *P* for trend | HR (95% CI) per score point |
|  | 0-1 | 2 | 3 | 4-5 |  |  |
| **Hypertension** |  |  |  |  |  |  |
| Total CVD |  |  |  |  |  |  |
| Cases/PYs | 1,323/61,999 | 1,693/84,725 | 887/51,708 | 101/6,740 |  |  |
| Model 1 | 1.00 | 0.86 (0.79, 0.92) | 0.74 (0.68, 0.81) | 0.67 (0.54, 0.82) | <0.01 | 0.87 (0.84, 0.90) |
| Model 2 | 1.00 | 0.89 (0.83, 0.96) | 0.80 (0.73, 0.88) | 0.72 (0.58, 0.88) | <0.01 | 0.90 (0.86, 0.93) |
| Ischemic Heart Disease |  |  |  |  |  |  |
| Cases/PYs | 312/65,279 | 371/89,308 | 167/54,247 | 31/6,988 |  |  |
| Model 1 | 1.00 | 0.78 (0.67, 0.91) | 0.59 (0.48, 0.71) | 0.89 (0.61, 1.28) | <0.01 | 0.83 (0.76, 0.89) |
| Model 2 | 1.00 | 0.83 (0.71, 0.97) | 0.63 (0.51, 0.77) | 0.90 (0.61, 1.31) | <0.01 | 0.85 (0.78, 0.93) |
| Ischemic Stroke |  |  |  |  |  |  |
| Cases/PYs | 518/64,694 | 676/88,170 | 326/53,605 | 34/6,995 |  |  |
| Model 1 | 1.00 | 0.87 (0.78, 0.98) | 0.70 (0.61, 0.81) | 0.57 (0.41, 0.81) | <0.01 | 0.84 (0.79, 0.89) |
| Model 2 | 1.00 | 0.91 (0.81, 1.03) | 0.77 (0.66, 0.90) | 0.64 (0.45, 0.92) | <0.01 | 0.88 (0.82, 0.93) |
| **Normotension** |  |  |  |  |  |  |
| Total CVD |  |  |  |  |  |  |
| Cases/PYs | 542/72,627 | 917/142,223 | 622/109,746 | 66/17,612 |  |  |
| Model 1 | 1.00 | 0.87 (0.78, 0.98) | 0.81 (0.71, 0.92) | 0.63 (0.48, 0.81) | <0.01 | 0.89 (0.85, 0.94) |
| Model 2 | 1.00 | 0.89 (0.80, 1.00) | 0.85 (0.74, 0.97) | 0.67 (0.51, 0.87) | <0.01 | 0.91 (0.86, 0.96) |
| Ischemic Heart Disease |  |  |  |  |  |  |
| Cases/PYs | 104/17,736 | 191/67,255 | 114/130,936 | 14/120,979 |  |  |
| Model 1 | 1.00 | 0.97 (0.76, 1.24) | 0.80 (0.60, 1.07) | 0.74 (0.42, 1.31) | 0.05 | 0.89 (0.79, 1.00) |
| Model 2 | 1.00 | 1.01 (0.79, 1.30) | 0.87 (0.64, 1.18) | 0.83 (0.46, 1.47) | 0.20 | 0.92 (0.81, 1.04) |
| Ischemic Stroke |  |  |  |  |  |  |
| Cases/PYs | 172/73,697 | 290/144,212 | 208/111,209 | 19/17,769 |  |  |
| Model 1 | 1.00 | 0.89 (0.73, 1.07) | 0.88 (0.70, 1.09) | 0.61 (0.37, 0.98) | 0.14 | 0.93 (0.85, 1.02) |
| Model 2 | 1.00 | 0.91 (0.75, 1.11) | 0.92 (0.73, 1.16) | 0.66 (0.41, 1.08) | 0.37 | 0.96 (0.87, 1.06) |

HRs were calculated in the Cox proportional hazards model. Model 1 was adjusted for sex; Model 2 was further adjusted for education level, marital status, household income, family history of heart attack or stroke, consumption of preserved vegetable, occupation, sedentary behavior, and use of antihypertensive drugs and duration of hypertension (for hypertensive population only).
